# Supplementary material for: Using instant messaging applications for consultations in the emergency department: A cross-sectional survey
Source: PLoS One. 2026 Apr 15;21(4):e0347028. doi: 10.1371/journal.pone.0347028 (PMC13082625; doi:10.1371/journal.pone.0347028)
Supplement: S1 File — Full survey instruments used for consultants and consultees assessing consultation practices and use of instant messaging applications in the emergency department. (DOCX) [file pone.0347028.s001.docx]

Appendix 1: ***Consultants Survey: This survey is specific to the consults for patients in the Emergency Department (not patients on floor)***

# Section 1

1. Age: Numerical
2. Gender:
   - Male
   - Female
3. Are you a:
   - Fellow (go to #4)
   - Resident (go to #5)
4. You are a/an fellow?
   - Endocrinology
   - Gastroenterology
   - Nephrology
   - Pulmonology/Critical Care
   - Adults Infectious Diseases
   - Pediatric Infectious Diseases
   - Adults Hematology/Oncology
   - Pediatrics Hematology/Oncology
   - Pediatric Intensive Care
   - Adults Cardiology
   - Pediatric Cardiology
   - Pediatric Neurology
   - Other:
5. You are a/an resident?
   - Anesthesiology
   - Dermatology
   - Radiology
   - Neurology
   - Pathology
   - Ophthalmology
   - Otorhinolaryngology
   - Psychiatry
   - Neurosurgery
   - General Surgery
   - Urology
   - Orthopedics
   - Plastics
   - Obstetrics and Gynecology
   - Other:
6. What year are you in?

|  | PGY1 |
| --- | --- |
|  | PGY2 |
|  | PGY3 |
|  | PGY4 |
|  | PGY5 |
|  | PGY6 |
|  | PGY7 |
|  | F1 |
|  | F2 |
|  | F3 |

1. How long is your shift when on call (in hours)? Numerical
2. Do you own a smartphone?
   - Yes
   - No
3. Was your smartphone lost or stolen in the last five years?
   - Yes
   - No
4. Is your smartphone ever used by someone else?
   - Yes
   - No
5. Do/Did consultees from ED contact you on your personal smartphone when you are on call/duty?
   - Yes
   - No
6. What method of consultation do you **prefer**?
   - Phone call on personal smartphone
   - Pager
   - Instant Messaging Applications (including WhatsApp and Webex)
7. How are you **actually** being contacted by consultees from the ED:
   - Workstation phone:
   - Pager:
   - Phone call from personal smart device:
   - Instant Messaging Applications (including WhatsApp and Webex):

Conditional question: what percentage of your consultations are conducted through each of the previously selected during the day call.

what percentage of your consultations are conducted through each of the previously selected during the night call.

**IF instants messaging application was not selected, survey will skip to section 3**

# Section 2

1. Which Instant Messaging Applications (WhatsApp, Webex…) do/did you get consulted on t**he most?**
   - Facebook Messenger
   - Viber
   - Telegram
   - Webex
   - WhatsApp
   - IMessage
   - Other:
2. On average, how many times do/did you get consulted by ED via Instant Messaging Applications (WhatsApp, Webex…) per call/duty?

Numerical

1. Which language do you get consulted with via Instant Messaging Applications (WhatsApp,

Webex…) the most?

- - English
  - Arabic
  - French

Chat language (Arabic written in English letters)

1. Do you get contacted for unofficial consultations via Instant Messaging Applications

(WhatsApp, Webex…) when on call?

- - Never (0% of the times)
  - Rarely (25% of the times)
  - Sometimes (50% of the times)
  - Often (75% of the times)
  - Always (100% of the times)

1. Have you ever consulted with your attending via Instant Messaging Applications

(WhatsApp, Webex…)?

- - Never (0% of the times)
  - Rarely (25% of the times)
  - Sometimes (50% of the times)
  - Often (75% of the times)
  - Always (100% of the times)

1. Have you received a consultation via Instant Messaging Applications (WhatsApp, Webex…)

when you were not on call/outside working hours?

- - Never (0% of the times)
  - Rarely (25% of the times)
  - Sometimes (50% of the times)
  - Often (75% of the times)
  - Always (100% of the times)

1. Which of the following formats do/did you receive consults by via Instant Messaging Applications (WhatsApp, Webex…)? (Please select all that apply)
   - Text
   - Image
   - Video
   - Voice message
   - Other:
2. Which of the following formats do you **trust** most when being consulted via Instant Messaging Applications (WhatsApp, Webex…)?
   - Text
   - Image
   - Video
   - Voice message
   - Other:
3. Which of the following formats do you **distrust** most when being consulted via Instant Messaging Applications (WhatsApp, Webex…)?
   - Text
   - Image
   - Video
   - Voice message
   - Other:
4. Do you think using Instant Messaging Applications (WhatsApp, Webex…) for consultation purposes is:

|  | Strongly  Disagree | Disagree | Neutral | Agree | Strongly  Agree |
| --- | --- | --- | --- | --- | --- |
| Fast | 1 | 2 | 3 | 4 | 5 |
| Easy | 1 | 2 | 3 | 4 | 5 |
| Reliable | 1 | 2 | 3 | 4 | 5 |
| Understandable | 1 | 2 | 3 | 4 | 5 |
| Accessible | 1 | 2 | 3 | 4 | 5 |
| Professional | 1 | 2 | 3 | 4 | 5 |
| Convenient | 1 | 2 | 3 | 4 | 5 |
| Distracting | 1 | 2 | 3 | 4 | 5 |

1. Have you ever faced connection problems or internet failure that hindered/delayed the consultation via Instant Messaging Applications (WhatsApp, Webex…)?
   - Never (0% of the times)
   - Rarely (25% of the times)
   - Sometimes (50% of the times)
   - Often (75% of the times)
   - Always (100% of the times)
2. Have you ever based your management/ diagnosis solely on a consultation done via Instant Messaging Applications (WhatsApp, Webex…) only without coming to see the patient?
   - Never (0% of the times)
   - Rarely (25% of the times)
   - Sometimes (50% of the times)
   - Often (75% of the times)
   - Always (100% of the times)
3. Do you think via Instant Messaging Applications (WhatsApp, Webex…) are adequate for consultation purposes without the consultant seeing the patient?
   - Extremely
   - Considerably
   - Moderately
   - Slightly
   - Not at all
4. Do you think that consulting via Instant Messaging Applications (WhatsApp, Webex…)

compromises the diagnosis?

- - Extremely
  - Considerably
  - Moderately
  - Slightly
  - Not at all

1. Do you or any of your colleagues have any experience of misdiagnosis with Instant Messaging Applications (WhatsApp, Webex…) consultation?
   - Never (0% of the times)
   - Rarely (25% of the times)
   - Sometimes (50% of the times)
   - Often (75% of the times)
   - Always (100% of the times)
2. Do you think consultation via Instant Messaging Applications (WhatsApp, Webex…) can cause any legal problems?
   - Yes
   - No
   - No idea
3. Do you receive the identity or personal information of the patients Instant Messaging Applications (WhatsApp, Webex…) consultation?
   - Never (0% of the times)
   - Rarely (25% of the times)
   - Sometimes (50% of the times)
   - Often (75% of the times)
   - Always (100% of the times)
4. Do you worry about the patients’ confidentiality when you receive consultations containing patients’ confidential information via Instant Messaging Applications (WhatsApp, Webex…)?
   - Yes
   - No
5. Do you usually have the patients’: (select all that apply)
   - Full name
   - Room number
   - MRN
6. How do you manage the patient information in your personal smartphone after finishing consultation?
   - I delete the confidential information as soon as I finish the consultation
   - I keep it in my phone for patient archiving and documentation
   - I keep it in my phone for any future legal problems
   - I do not have any special attention for patient information
   - I don’t receive consults with patients confidential information

# Section 3

|  | Strongly Disagree | Disagree | Neutral | Agree | Strongly Agree |
| --- | --- | --- | --- | --- | --- |
| 34) The current consultation process is adequate. | 1 | 2 | 3 | 4 | 5 |
| 35) The current consultation process should be improved/more organized. | 1 | 2 | 3 | 4 | 5 |
| 36) It is necessary to develop an electronic platform or an application exclusively for consultation. | 1 | 2 | 3 | 4 | 5 |

1. Write your concerns about the current consultation process.
2. Write your suggestions to improve the consultation process.

We are considering the potential for Phase 2 of this study, which would involve conducting semi-structured interviews regarding your experience with the consultation process. If you're interested in participating, a separate popup window will appear to collect your email address. Please note that the email address you provide will be stored independently and will not be associated or linked to your previous responses in any way.

1. **Are you interested in participating?**
   - Yes
   - No

**Appendix 4*: Consultee Survey***

# Section 1**:**

1. Age: Numerical
2. Gender:
   - Male
   - Female
3. Which program are you in now?
   - Med IV
   - Emergency Medicine
   - Moonlighter (House Physicians)
   - Pediatrics
   - Internal Medicine
   - Family Medicine
   - Otorhinolaryngology
   - Anesthesiology
   - Radiology
   - Obstetrics and Gynecology
   - Psychiatry
   - Other:
4. Which postgraduate year are you in?
   - Med IV
   - PGY 1
   - PGY 2
   - PGY 3
   - PGY 4
   - PGY 5
5. How long is your ED shift (in hours)? Numerical
6. Do you own a smartphone?
   - Yes
   - No
7. Was your smartphone lost or stolen in the last five years?
   - Yes
   - No
8. Is your smartphone sometimes used by someone else?
   - Yes
   - No
9. Do/did you use your smartphone during your ED shifts?
   - Yes
   - No
10. What method of contacting consultants do you **prefer**?
    - Workstation phone
    - Phone call from personal smartphone
    - Pager
    - Instant Messaging Applications (WhatsApp, Webex…)
11. What routes of contacting consultants **do you actually use** (select all that apply):
    - Workstation phone:
    - Pager:
    - Phone call from personal smart device:
    - Instant Messaging Applications (WhatsApp, Webex…):

Conditional question: what percentage of your consultations are conducted through each of the previously selected during the day shift.

What percentage of your consultations are conducted through each of the previously selected during the night shift.

**If instants messaging application was not selected, survey will skip to section 3**

# Section 2

1. Which Instant Messaging Applications (WhatsApp, Webex…) do/did you use **the most** when contacting consultants?
   - Facebook Messenger
   - Viber
   - Telegram
   - Webex
   - WhatsApp
   - iMessage
   - Other:
2. On average, how many consultations do you do using Instant Messaging Applications (WhatsApp, Webex…) per shift?

Numerical:

1. Which language do you use the most when contacting consultants via Instant Messaging Applications (WhatsApp, Webex…)?
   - English
   - Arabic
   - French
   - Chat language (Arabic written in English letters)
2. Do you contact consultants on Instant Messaging Applications (WhatsApp, Webex…) for unofficial consultations when on shift?
   - Never (0% of the times)
   - Rarely (25% of the times)
   - Sometimes (50% of the times)
   - Often (75% of the times)
   - Always (100% of the times)
3. Which of the following formats do/did you use for consultation purposes on Instant Messaging Applications (WhatsApp, Webex…)? (Please select all that apply)
   - Text
   - Image
   - Video
   - Voice message
   - Other:
4. Which of the following formats do you **trust** most when consulting on Instant Messaging Applications (WhatsApp, Webex…)?
   - Text
   - Image
   - Video
   - Voice message
   - Other:
5. Which of the following formats do you **distrust** most when consulting on Instant Messaging Applications (WhatsApp, Webex…)?
   - Text
   - Image
   - Video
   - Voice message
   - Other:
6. Do you think using Instant Messaging Applications (WhatsApp, Webex…) for consultation purposes is:

|  | Strongly  Disagree | Disagree | Neutral | Agree | Strongly  Agree |
| --- | --- | --- | --- | --- | --- |
| Fast | 1 | 2 | 3 | 4 | 5 |
| Easy | 1 | 2 | 3 | 4 | 5 |
| Reliable | 1 | 2 | 3 | 4 | 5 |
| Understandable | 1 | 2 | 3 | 4 | 5 |
| Accessible | 1 | 2 | 3 | 4 | 5 |
| Professional | 1 | 2 | 3 | 4 | 5 |
| Convenient | 1 | 2 | 3 | 4 | 5 |
| Distracting | 1 | 2 | 3 | 4 | 5 |

1. Have you ever faced connection problems or internet failure that hindered/delayed the consultation on Instant Messaging Applications (WhatsApp, Webex…) during your shift?
   - Never (0% of the times)
   - Rarely (25% of the times)
   - Sometimes (50% of the times)
   - Often (75% of the times)
   - Always (100% of the times)
2. Have you ever based your management solely on a consultation finalized via Instant Messaging Applications (WhatsApp, Webex…) without the consultant coming to see the patient?
   - Never (0% of the times)
   - Rarely (25% of the times)
   - Sometimes (50% of the times)
   - Often (75% of the times)
   - Always (100% of the times)
3. Do you think Instant Messaging Applications (WhatsApp, Webex…) are adequate for consultation purposes without the consultant seeing the patient?
   - Extremely
   - Considerably
   - Moderately
   - Slightly
   - Not at all
4. Do you think that consulting via Instant Messaging Applications (WhatsApp, Webex…) compromises the diagnosis?
   - Extremely
   - Considerably
   - Moderately
   - Slightly
   - Not at all
5. Do you or any of your colleagues have any experience of misdiagnosis with Instant Messaging Applications (WhatsApp, Webex…) consultations?
   - Never (0% of the times)
   - Rarely (25% of the times)
   - Sometimes (50% of the times)
   - Often (75% of the times)
   - Always (100% of the times)
6. Do you think consultation via Instant Messaging Applications (WhatsApp, Webex…) can cause any legal problems?
   - Yes
   - No
   - No idea
7. Do you use the identity or personal information of the patients when using Instant Messaging Applications (WhatsApp, Webex…)?
   - Never (0% of the times)
   - Rarely (25% of the times)
   - Sometimes (50% of the times)
   - Often (75% of the times)
   - Always (100% of the times)
8. Do you worry about the patients’ confidentiality when you send consultations containing

patients’ confidential information via Instant Messaging Applications (WhatsApp, Webex…)?

- - Never (0% of the times)
  - Rarely (25% of the times)
  - Sometimes (50% of the times)
  - Often (75% of the times)
  - Always (100% of the times)

1. Do you usually use the patients’: (select all that apply)
   - Full name
   - Room number
   - MRN
2. Do you take the patient’s consent before sharing any identifying information with the

consultants via Instant Messaging Applications (WhatsApp, Webex…)?

- - Yes
  - No
  - There is no need for consent in such situations

1. How do you manage the patient information in your personal smartphone after finishing consultation?
   - I delete the confidential information as soon as I finish the consultation
   - I keep it in my phone for patient archiving and documentation
   - I keep it in my phone for any future legal problems
   - I do not have any special attention for patient information
   - I do not share the patients confidential information in the first place

# Section 3

|  | Strongly Disagree | Disagree | Neutral | Agree | Strongly Agree |
| --- | --- | --- | --- | --- | --- |
| 31) The current consultation process is adequate. | 1 | 2 | 3 | 4 | 5 |
| 32) The current consultation process should be improved/more organized. | 1 | 2 | 3 | 4 | 5 |
| 33) It is necessary to develop an electronic platform or an application exclusively for consultation. | 1 | 2 | 3 | 4 | 5 |

1. Write your concerns about the current consultation process.
2. Write your suggestions to improve the consultation process.

We are considering the potential for Phase 2 of this study, which would involve conducting semi-structured interviews regarding your experience with the consultation process. If you're interested in participating, a separate popup window will appear to collect your email address. Please note that the email address you provide will be stored independently and will not be associated or linked to your previous responses in any way.

1. **Are you interested in participating?**
   - Yes
   - No
