# Supplementary material for: Using instant messaging applications for consultations in the emergency department: A cross-sectional survey
Source: PLoS One. 2026 Apr 15;21(4):e0347028. doi: 10.1371/journal.pone.0347028 (PMC13082625; doi:10.1371/journal.pone.0347028)
Supplement: Attachment — Submitted filename: PONE-D-25-46511 Using Instant Messaging App study.pdf [file pone.0347028.s003.pdf]

PLOS ONE

Using Instant Messaging Applications for Consultations in the Emergency Department: A cross-sectional survey

--Manuscript Draft--

**Manuscript Number:**	PONE-D-25-46511
**Article Type:**	Research Article
**Full Title:**	Using Instant Messaging Applications for Consultations in the Emergency Department: A cross-sectional survey
**Short Title:**	Instant Messaging Study
**Corresponding Author:**	Afif Mufarrij, MD<br>American University of Beirut Medical Center<br>Beirut, LEBANON
**Keywords:**	Emergency Department, Instant Messaging Applications, WhatsApp, Telemedicine, Healthcare communication, Cross-sectional survey
**Abstract:**	Study Objective: This study aims to investigate how residents and consultants use Instant Messaging Applications (IMAs) for consultations in the Emergency Department (ED), exploring their attitudes towards these applications and identifying areas for improvement in communication practices. Methods: A cross-sectional survey was conducted among residents, medical students, and consultants at the American University of Beirut Medical Center, Lebanon. Participants completed an online questionnaire assessing their demographics, consultation patterns, IMAs usage, and opinions on consultation modalities. The recruitment period was from 13/11/2023 to 08/11/2024. Results: The study included 120 participants, (65 consultants and 55 consultees). Consultants were significantly older than consultees (28.7 ± 3.4 vs. 26.6 ± 1.91 years, p < 0.001). While all consultees were residents, 64.6% of consultants were residents, and 35.4% were fellows (p < 0.001). All participants reported using smart phones (SP) when on call for consultation (100.0%). IMAs were the most preferred method of consultation (48.3%), followed by phone calls via SP (32.5%) among all participants. Consultants favored SP calls (49.2% vs. 12.7%, p < 0.001), while consultees preferred workstation phone calls (30.9% vs. 0%, p < 0.001). WhatsApp was the dominant IMA (98.9%). Consultants and consultees perceived IMAs as fast, easy, and accessible, though consultants found them more distracting (3.3 ± 1.4 vs. 2.5 ± 1.3, p = 0.007). While 63.7% acknowledged potential legal issues, IMAs remained a widely used consultation tool. Conclusion: The findings highlight the need for more structured, secure digital solutions to optimize communication practices within healthcare teams. We propose the development of an AI-generated application tailored for hospital consultations, ensuring compliance with privacy regulations, and improving the efficiency and safety of the consultation process.
**Order of Authors:**	Sarah Abdul Nabi<br>Souraya Arabi<br>Hind Anan<br>Hani Tamim<br>Jean Marie Al Semaani<br>Maha Makki<br>Afif Mufarrij, MD
**Additional Information:**	
**Question**	**Response**
**Financial Disclosure**<br><br>Enter a financial disclosure statement that describes the sources of funding for the	The author(s) received no specific funding for this work.

work included in this submission. Review the submission guidelines for detailed requirements. View published research articles from *PLOS ONE* for specific examples.<br><br>This statement is required for submission and **will appear in the published article** if the submission is accepted. Please make sure it is accurate.<br><br>**Funded studies**<br>Enter a statement with the following details:<br>• Initials of the authors who received each award<br>• Grant numbers awarded to each author<br>• The full name of each funder<br>• URL of each funder website<br>• Did the sponsors or funders play any role in the study design, data collection and analysis, decision to publish, or preparation of the manuscript?<br><br>Did you receive funding for this work?	
**Competing Interests**<br><br>Use the instructions below to enter a competing interest statement for this submission. On behalf of all authors, disclose any competing interests that could be perceived to bias this work—acknowledging all financial support and any other relevant financial or non-financial competing interests.<br><br>This statement is required for submission and **will appear in the published article** if the submission is accepted. Please make sure it is accurate and that any funding sources listed in your Funding Information later in the submission form are also declared in your Financial Disclosure statement.<br><br>View published research articles from *PLOS ONE* for specific examples.	The authors have declared that no competing interests exist

**NO authors have competing interests**<br><br>Enter: *The authors have declared that no competing interests exist.*<br><br>**Authors with competing interests**<br><br>Enter competing interest details beginning with this statement:<br><br>*I have read the journal's policy and the authors of this manuscript have the following competing interests: [insert competing interests here]*<br><br>* typeset	
**Ethics Statement**<br><br>Enter an ethics statement for this submission. This statement is required if the study involved:<br><br>• Human participants<br>• Human specimens or tissue<br>• Vertebrate animals or cephalopods<br>• Vertebrate embryos or tissues<br>• Field research<br><br>Write "N/A" if the submission does not require an ethics statement.<br><br>General guidance is provided below. Consult the submission guidelines for detailed instructions. **Make sure that all information entered here is included in the Methods section of the manuscript.**	Approval was granted by the Institutional Review Board under protocol number: SBS-2023-0009

Powered by Editorial Manager® and ProduXion Manager® from Aries Systems Corporation

**Format for specific study types**<br><br>**Human Subject Research (involving human participants and/or tissue)**<br>• Give the name of the institutional review board or ethics committee that approved the study<br>• Include the approval number and/or a statement indicating approval of this research<br>• Indicate the form of consent obtained (written/oral) or the reason that consent was not obtained (e.g. the data were analyzed anonymously)<br><br>**Animal Research (involving vertebrate animals, embryos or tissues)**<br>• Provide the name of the Institutional Animal Care and Use Committee (IACUC) or other relevant ethics board that reviewed the study protocol, and indicate whether they approved this research or granted a formal waiver of ethical approval<br>• Include an approval number if one was obtained<br>• If the study involved *non-human primates*, add *additional details* about animal welfare and steps taken to ameliorate suffering<br>• If anesthesia, euthanasia, or any kind of animal sacrifice is part of the study, include briefly which substances and/or methods were applied<br><br>**Field Research**<br><br>Include the following details if this study involves the collection of plant, animal, or other materials from a natural setting:<br>• Field permit number<br>• Name of the institution or relevant body that granted permission	
**Data Availability**<br><br>Authors are required to make all data underlying the findings described fully available, without restriction, and from the time of publication. PLOS allows rare exceptions to address legal and ethical concerns. See the PLOS Data Policy and FAQ for detailed information.	Yes - all data are fully available without restriction

A Data Availability Statement describing where the data can be found is required at submission. Your answers to this question constitute the Data Availability Statement and **will be published in the article**, if accepted.<br><br>**Important:** Stating 'data available on request from the author' is not sufficient. If your data are only available upon request, select 'No' for the first question and explain your exceptional situation in the text box.<br><br>Do the authors confirm that all data underlying the findings described in their manuscript are fully available without restriction?	
**Describe where the data may be found in full sentences. If you are copying our sample text, replace any instances of XXX with the appropriate details.**<br><br>• If the data are **held or will be held in a public repository**, include URLs, accession numbers or DOIs. If this information will only be available after acceptance, indicate this by ticking the box below. For example: *All XXX files are available from the XXX database (accession number(s) XXX, XXX.).*<br>• If the data are all contained **within the manuscript and/or Supporting Information files**, enter the following: *All relevant data are within the manuscript and its Supporting Information files.*<br>• If neither of these applies but you are able to provide **details of access elsewhere**, with or without limitations, please do so. For example:<br><br>*Data cannot be shared publicly because of [XXX]. Data are available from the XXX Institutional Data Access / Ethics Committee (contact via XXX) for researchers who meet the criteria for access to confidential data.*<br><br>*The data underlying the results presented in the study are available from (include the name of the third party*	Data is stored and locked both in paper and digitally, with access given only to corresponding author.

and contact information or URL).<br>• This text is appropriate if the data are owned by a third party and authors do not have permission to share the data.<br><br>* typeset	
Additional data availability information:	

Powered by Editorial Manager® and ProduXion Manager® from Aries Systems Corporation

Manuscript Click here to access/download;Manuscript;1. Manuscript.docx

TITLE: Using Instant Messaging Applications for Consultations in the Emergency Department: A cross-sectional survey

Authors: Sarah Abdul-Nabi[1*], MD, Souraya Arabi, MD[1*], Hind Anan, MD[2], Hani Tamim, PhD[3,4], Jean-Marie Al Semaani[2], Maha Makki, MSc[3], Afif-Jean Mufarrij, MD[1]

*Both authors contributed equally and should be considered as co-first

Affiliations
[1]Department of Emergency Medicine, American University of Beirut, Beirut, Lebanon
[2]Faculty of Medicine, American University of Beirut, Beirut, Lebanon
[3]Clinical Research Institute, American University of Beirut, Beirut, Lebanon
[4]College of Medicine, Alfaisal University, Riyadh, Saudi Arabia

Corresponding Author:
Afif Jean Mufarrij, MD
Address: P.O.Box 11-0236, Riad El-Solh / Beirut 1107 2020, Beirut, Lebanon.
Tel: 00961-1-350000 ext. 7876
Email: am66@aub.edu.lb

Word count: 4696

Author Contribution: AM and HA were responsible for conceptualization and study design. HT and MM worked on data cleaning and statistical analysis. SAN, SA and JA were responsible for drafting of the manuscript as well as data collection, ensuring the accuracy and completeness of the dataset. AM provided supervision, project administration and critical revisions. All authors reviewed and approved the final version of the manuscript.

ABSTRACT

Study Objective: This study aims to investigate how residents and consultants use Instant Messaging Applications (IMAs) for consultations in the Emergency Department (ED), exploring their attitudes towards these applications and identifying areas for improvement in communication practices.

Methods: A cross-sectional survey was conducted among residents, medical students, and consultants at the American University of Beirut Medical Center, Lebanon. Participants completed an online questionnaire assessing their demographics, consultation patterns, IMAs usage, and opinions on consultation modalities. The recruitment period was from 13/11/2023 to 08/11/2024.

Results: The study included 120 participants, (65 consultants and 55 consultees). Consultants were significantly older than consultees (28.7 ± 3.4 vs. 26.6 ± 1.91 years, $p < 0.001$). While all consultees were residents, 64.6% of consultants were residents, and 35.4% were fellows ($p < 0.001$). All participants reported using smart phones (SP) when on call for consultation (100.0%). IMAs were the most preferred method of consultation (48.3%), followed by phone calls via SP (32.5%) among all participants. Consultants favored SP calls (49.2% vs. 12.7%, $p < 0.001$), while consultees preferred workstation phone calls (30.9% vs. 0%, $p < 0.001$). WhatsApp was the dominant IMA (98.9%). Consultants and consultees perceived IMAs as fast, easy, and accessible, though consultants found them more distracting (3.3 ± 1.4 vs. 2.5 ± 1.3, $p = 0.007$). While 63.7% acknowledged potential legal issues, IMAs remained a widely used consultation tool.

Conclusion: The findings highlight the need for more structured, secure digital solutions to optimize communication practices within healthcare teams. We propose the development of an AI-generated application tailored for hospital consultations, ensuring compliance with privacy regulations, and improving the efficiency and safety of the consultation process.

Introduction

The integration of mobile devices into various professional fields, including healthcare, has transformed medical practice and education. Currently, the majority of medical workers own smartphones (SP) they use often in their clinical routines(1, 2). In fact, a study by Boruff et al. revealed that 93.6% of medical students and residents accessed medical resources and applications on their mobile devices(2). While only 83 healthcare applications were documented back in 2011(3), continuous advancements in technology have magnified this number, highlighting the growing reliance on mobile tools in healthcare practice. Smartphone use by healthcare providers significantly enhances clinical decision-making, patient outcomes, and satisfaction, illustrating its potential to improve healthcare quality and efficiency (1, 3-5). Physicians frequently utilize smartphones for oral (56.4%) and written (38.9%) communication, as they find them more efficient than the traditional pagers (1, 9). Studies have shown that mobile devices facilitate faster and more reliable communication, reducing delays in decision-making, and minimizing the risk of medical errors(6-9). In particular, WhatsApp has gained widespread use in Emergency Departments (EDs) for text messaging, voice notes, and multimedia sharing. It has considerably

reduced the patients’ length of stay, with a median difference of 30 minutes compared to the standard telephone communication(10).

At the American University of Beirut Medical Center (AUBMC), mobile devices are widely adopted by healthcare providers. All ED personnel owned a mobile device, with most reporting using medical applications in their clinical practice(11). The majority agreed that mobile devices improved communication and patient care, although fewer felt these tools enhanced teamwork within the department (11). Instant Messaging Applications (IMAs) such as WhatsApp were widely adopted, though it remains difficult to clearly differentiate between clinical and personal use(11, 12).

Given the critical role of communication in the ED, where response rates and time are essential, this study aims to investigate how IMAs, particularly WhatsApp, is used when consulting with medical professionals, exploring their attitudes towards these applications, and identifying areas for improvement in communication practice.

Methodology

Study Design and Setting

This is a cross-sectional study conducted at the ED at AUBMC. AUBMC is an academic tertiary care hospital, handling approximately 55,000 ED visits each year. The ED is staffed by a combination of physicians trained in Emergency Medicine (EM) and experienced practitioners with substantial EM expertise. The EM Residency Program at AUBMC was started in June 2012 as a four-year training program, with an average of five residents per year. In addition to EM residents, residents from other specialties as well as medical students in their fourth year also rotate through the ED as part of their training.

Consultations play a crucial role in patient management in the ED. The ED conducts approximately 18,300 consultations annually, averaging around 50 per day, with varying levels of acuity, ranging from minor injuries to critical conditions requiring immediate intervention. Previously, AUBMC relied on pagers for communication, but later transitioned to the Webex system for improved efficiency. Since AUBMC provides free Wi-Fi, the use of smartphones has become more convenient and cost-free for staff.

The consultation process begins with the consultee evaluating the patient and presenting the case to the ED attending physician, who oversees the initial workup. If a specialist consultation is required, the on-call consultant is contacted. The consultant then comes to the ED, assesses the patient, and provides the consultee with the most appropriate plan after talking to the attending physician (figure 1).

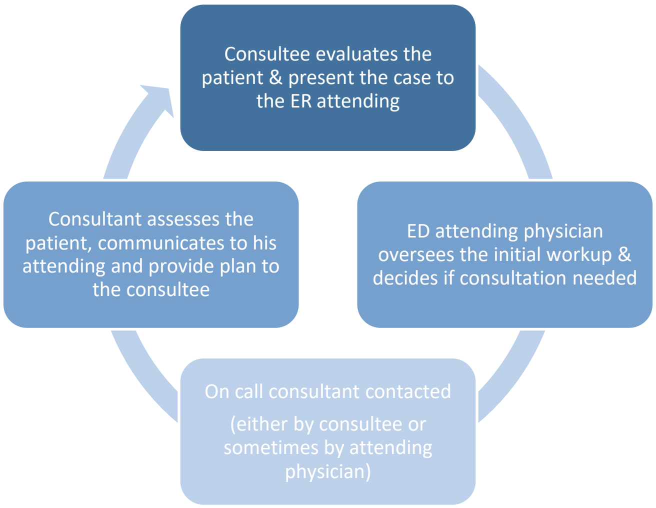

Figure.1: Consultation process flow chart

As per AUBMC policy regarding consultation response to calls from the Emergency Department, the ED resident under the supervision of the ED Attending typically initiates clinical consultations. To make the consultation official, a consultation order must be placed in the EPIC system. The ED team contacts the on-call resident or fellow from the consultant service. If there is no response after two contact attempts within 10 minutes, the ED Attending escalates to the Consultant Service Attending-On-Call, who becomes responsible for ensuring a prompt response.

Selection of Participants

A cross-sectional survey was sent by email to all residents, fourth year medical students, and consultants at AUBMC. It was initially piloted and reviewed by 2–3 consultants and consultees to ensure clarity and relevance before it was distributed. The survey was in English, as all participants demonstrate English proficiency prior to their positions. It explored participants demographics, the consultation process, methods of communication with consultants, and perspectives on patient privacy. Two tailored survey versions were used: Consultees' Survey (Designed for medical students and residents working in the ED) and Consultants' Survey (Addressed to residents and fellows serving as consultants). Both versions followed similar themes with minor role-specific differences. Approval was granted by the Institutional Review Board under protocol number: SBS-2023-0009. This study poses minimal risk to participants, as all survey responses are anonymous and confidential. Participants provided informed online consent before completing the survey. Participation was voluntary, with no identifying information collected. The recruitment period was from 13/11/2023 to 08/11/2024. A copy of the questionnaire developed for this study has been provided as Supplementary File 1.

The study population includes two groups: Consultees and Consultants

Consultees (covering ED shifts)	Consultants
Residents (Emergency Medicine residents and moonlighters, as well as residents rotating in the ED from various specialties:<br>Pediatrics, Internal Medicine, Family Medicine, Otorhinolaryngology, Anesthesiology, Radiology, Obstetrics and Gynecology, Psychiatry and Medical Students.	- Residents: Specialties including Anesthesiology, Dermatology, Radiology, Neurology, Ophthalmology, Otorhinolaryngology, Psychiatry, Surgery (Neurosurgery, General Surgery, Critical Care, Urology, Orthopedics, Plastics), and Obstetrics and Gynecology)<br>- Clinical fellows consulted by ED residents, moonlighters, or students. Subspecialties such as Endocrinology, Gastroenterology, Nephrology, Pulmonology, Infectious Diseases (adult & pediatric), Hematology/Oncology (adult & pediatric), Intensive Care (adult & pediatric), Cardiology (adult & pediatric), and Pediatric Neurology).

Inclusion criteria for consultees are all medical students and residents as well as moonlighters covering shifts in the ED. Inclusion criteria for consultants are all residents and clinical fellows

consulted by ED residents. There were no exclusion criteria, participation was open to all eligible individuals who consent to take part

In the study, participants are categorized as follow:

- Surgical specialties: General Surgery, Orthopedics, Neurosurgery, OBGYN, Ophthalmology, Otorhinolaryngology
- Acute non-surgical specialties with potential procedures: Radiology, Neurology, Anesthesia, Plumo/ICU, Cardiology, Emergency Medicine
- Acute non-surgical specialties with NO potential procedures: Psychiatry, Pathology, Family Medicine, Nephrology, Gastroenterology, Infectious disease, Endocrinology, Pediatrics, Internal medicine
- Junior Residents: House Physician, Post graduate year (PGY) 1 & PGY2
- Senior Residents: PGY3,4,5,6

Data Collection and Analysis

Data collection was extracted through SPSS. The Statistical Package for the Social Sciences (SPSS) version 25.0 was used for data cleaning, management, and analysis. Descriptive statistics were presented as mean ± standard deviation (SD) for continuous variables. Categorical data were reported as frequencies and percentages. In the bivariate analysis, the relationships between participant roles (consultant vs consultee) and other categorical variables was analyzed using Pearson's Chi-square **test.** Whereas Student's t-test was used for the association with continuous variables. The statistical significance was set at p-value < 0.05**.**

Results

TABLE 1: Demographics and Professional Characteristics (Residency Program and Level) of Consultants and Consultees

		Total N=120	Consultant N=65	Consultees N=55	P-value
**Gender**	Male	48 (40.0%)	29 (44.6%)	19 (34.5%)	0.26
**Age**	Mean ± SD	27.7 ± 3.0	28.7 ± 3.4	26.6 ± 1.9	<0.001
**Position**	Resident	97 (80.8%)	42 (64.6%)	55 (100.0%)	<0.001
	Fellow	23 (19.2%)	23 (35.4%)	0 (0.0%)	
**Program**	Surgical specialties*	16 (13.7%)	16 (25.0%)	0 (0.0%)	<0.001
	Acute non-surgical specialties with potential procedures**	43 (37.8%)	24 (37.5%)	19 (35.8%)	
	Acute non-surgical specialties with NO potential procedures***	44 (37.6%)	24 (37.5%)	20 (37.7%)	

	Students	14 (12.0%)	0 (0.0%)	14 (26.4%)	
**Year**	Junior Residents^	38 (31.7%)	15 (23.1%)	23 (41.8%)	<0.001
	Senior Residents^^	55 (45.8%)	37 (56.9%)	18 (32.7%)	
	Fellows	13 (10.8%)	13 (20.0%)	0 (0.0%)	
	Students	14 (11.7%)	0 (0.0%)	14 (25.5%)	

*Surgical specialties: General Surgery, Orthopedics, Neurosurgery, OBGYN, Ophthalmology, Otorhinoloryngology
** Acute non-surgical specialties with potential procedures: Radiology, Neurology, Anesthesia, Plumo/ICU, Cardiology, Emergency Medicine
*** Acute non-surgical specialties with NO potential procedures: Psychiatry, Pathology, Family Medicine, Nephrology, Gastroenterology, Infectious disease, Endocrinology, Pediatrics, Internal medicine
^Junior Residents: House Physician, PGY1 & PGY2
^^Senior Residents: PGY3,4,5,6

Table 1 presents the Demographics and Professional Characteristics (Residency Program and Level) of Consultants and Consultees. The study included a total of 120 participants, 40% of which were male. These consisted of 65 consultants and 55 consultees. The mean age of the consultants was significantly higher than that of the consultees (28.7 ± 3.4 vs 26.6 ± 1.9 years, $p < 0.001$). The distribution of participants across residency programs also showed significant variation ($p<0.001$). While 25.0% of consultants belonged to surgical specialties, the remaining participants were distributed across acute non-surgical specialties with potential procedures (37.5% consultants vs. 35.8% consultees) and acute non-surgical specialties with no potential procedures (37.5% consultants vs. 37.7% consultees). In terms of residency level, junior residents constituted 41.8% of consultees, which is significantly higher than that of consultants (41.8 vs 23.1%, $p < 0.001$). Conversely, senior residents made up a greater proportion of consultants (56.9%) compared to consultees (32.7%). 20% of consultants were fellows and students accounted for 25.5% of consultee.

TABLE 2: General Information Related to smartphones (SP) Usage and Consultation Preferences Among Consultants and Consultees

		**Total N=120**	**Consultant N=65**	**Consultees N=55**	**P-value**
**Ownership of a SP**	Yes	120 (100.0%)	65 (100.0%)	55 (100.0%)	-
**Use of SP when on call/duty**	Yes	120 (100.0%)	65 (100.0%)	55 (100.0%)	-
**Use of one’s SP by someone else**	Yes	20 (16.7%)	7 (10.8%)	13 (23.6%)	0.06
**Preferred method of consultation**	IMA	58 (48.3%)	29 (44.6%)	29 (52.7%)	<0.001
	Phone call on SP	39 (32.5%)	32 (49.2%)	7 (12.7%)	
	Workstation phone call	17 (14.2%)	0 (0.0%)	17 (30.9%)	
	Pager	6 (5.0%)	4 (6.2%)	2 (3.6%)	
**Shift duration when on call (in hours)**	Mean ± SD	16.6 ± 9.1	23.1 ± 7.7	9.0 ± 1.9	<0.001

Table 2 presents General Information Related to SP Usage and Consultation Preferences Among Consultants and Consultees. All (100%) participants reported having a SP and using it while on call or duty. The preferred method of consultation for consultees was IMAs (48.3%, $p < 0.001$) while consultants preferred phone calls on SP (49.2%). In fact, both groups favored using IMAs like WhatsApp, with 44.6% of consultants and 52.7% of consultees indicating this preference. While 30.9% of consultees preferred using workstation phone calls, none of the consultants selected this method. On the other hand, consultants preferred using a phone call on their personal smartphones (49.2%), significantly more than consultees (12.7%, $p<0.001$). A significant difference was found in the shift duration when on call where consultants had a longer shift than consultees (23.1 ± 7.7 hours vs. 9.0 ± 1.9 hours, $p < 0.001$).

TABLE 3: Consultation Patterns among Consultants and Consultees during Day and Night Call

		Total N=120	Consultant N=65	Consultees N=55	P-value
**Pager**	Day call	28 (23.5%)	10 (15.6%)	18 (32.7%)	0.03
	Night call	24 (20.0%)	8 (12.3%)	16 (29.1%)	0.02
**Workstation phone call**	Day call	82 (68.3%)	37 (56.9%)	45 (81.8%)	0.003
	Night call	76 (63.3%)	32 (49.2%)	44 (80.0%)	<0.001
**Phone call from SP**	Day call	93 (77.5%)	53 (81.5%)	40 (72.7%)	0.25
	Night call	90 (75.0%)	55 (84.6%)	35 (63.6%)	0.008
**IMA**	Day call	96 (80.0%)	53 (81.5%)	43 (78.2%)	0.65
	Night call	75 (62.5%)	38 (58.5%)	37 (67.3%)	0.32

Table 3 presents Consultation Patterns among Consultants and Consultees During Day and Night Calls. For pager use, consultees were significantly more likely to use pagers compared to consultants during both day calls (32.7% vs. 15.6%, $p = 0.03$) and night calls (29.1% vs. 12.3%, $p = 0.02$). Similarly, the use of workstation phone calls differed significantly between the two groups. During day calls, consultees relied more on workstation phone calls compared to consultants (81.8% vs 56.9%, $p = 0.003$). This difference was even more pronounced during night calls, where 80.0% of consultees used workstation phone calls compared to only 49.2% of consultants ($p < 0.001$). Regarding phone calls made via SP, a significant difference was observed during night calls, with a higher proportion of consultants using this method compared to consultees (84.6% vs. 63.6%, $p = 0.008$). Although the overall use of IMA did not significantly differ between consultants and consultees during day or night calls.

TABLE 4: Usage Patterns and Frequency of IMA for Consultations among Consultants and Consultee

		Total N=120	Consultant N=65	Consultees N=55	P-value
**Most used IMA**	WhatsApp	90 (98.9%)	46 (97.9%)	44 (100.0%)	0.33
	Other	1 (1.1%)	1 (2.1%)	0 (0.0%)	

**Average number of IMA consultations per call/shift**	Mean ± SD	5.7 ± 6	4.9 ± 4.2	6.4 ± 7.4	0.23
**Language used via IMA**	English	73 (80.2%)	36 (76.6%)	37 (84.1%)	0.33
	Arabic	3 (76.6%)	3 (6.4%)	0 (0.0%)	
	Chat language (Arabic written in English letters)	15 (16.5%)	8 (17%)	7 (15.9%)	

Table 4 presents Usage Patterns and Frequency of IMA for Consultations among Consultants and Consultees. WhatsApp was the most frequently used Instant Messaging Application IMA for consultations, with nearly all participants (98.9%) relying on it, and no significant differences observed between consultants and consultees (p=0.33). The average number of consultations conducted via IMA per call or duty was slightly higher among consultees compared to consultants though this difference was not statistically significant (6.45 ± 7.42 vs. 4.94 ± 4.21, p = 0.23). Regarding language use in IMA consultations, English was the predominant language for both groups (80.2%), with no significant variation between consultants and consultees (p = 0.33).

TABLE 5: Physicians (Consultants and Consultees) and medical student opinion regarding the use of IMA for consultations

			Total N=120	Consultant N=65	Consultees N=55	P-value
**Most USED method**		Text Message	87 (95.6%)	46 (97.9%)	41 (93.2%)	0.28
		Image	48 (52.7%)	20 (42.6%)	28 (63.6%)	0.04
		Video	11 (12.1%)	6 (12.8%)	5 (11.4%)	0.84
		Voice Message	75 (82.4%)	42 (89.4%)	33 (75.0%)	0.07
**Most TRUSTED method**		Text Message	64 (70.3%)	35 (74.5%)	29 (65.9%)	0.37
		Image	16 (17.6%)	14 (29.8%)	2 (4.5%)	0.002
		Video	3 (3.3%)	3 (6.4%)	0 (0.0%)	0.24
		Voice Message	22 (26.8%)	19 (40.4%)	3 (8.6%)	0.001
**Most DISTRUSTED method**		Text Message	16 (17.6%)	8 (17.0%)	8 (18.2%)	0.88
		Image	22 (24.2%)	15 (31.9%)	7 (15.9%)	0.07
		Video	12 (13.2%)	8 (17.0%)	4 (9.1%)	0.26
		Voice Message	40 (44.0%)	22 (46.8%)	18 (40.9%)	0.57
**Consultation Request through IMA causes any legal problems**		Yes	58 (63.7%)	31 (66.0%)	27 (61.4%)	0.25
For the below questions: Answers range from 0% (Never) to 100% (Always)						
**Consultation request through IMA is:**	**Fast**	Percentage	84.4 ± 23.6	84.2 ± 26.0	84.6 ± 21.0	0.93
	**Easy**	Percentage	87.9 ± 24.0	87.8 ± 24.9	88.1 ± 23.2	0.95
	**Reliable**	Percentage	69.8 ± 26.2	67.5 ± 28.5	72.2 ± 23.6	0.40
	**Understandable**	Percentage	75.8 ± 24.8	73.4 ± 25.8	78.4 ± 23.9	0.34
	**Accessible**	Percentage	88.5 ± 19.5	87.8 ± 20.1	89.2 ± 19.0	0.73
	**Professional**	Percentage	51.1 ± 28.1	50.0 ± 31.3	52.3 ± 24.6	0.70
	**Convenient**	Percentage	80.8 ± 24.7	80.8 ± 22.8	80.7 ± 26.9	0.97
	**Distracting**	Percentage	48.9 ± 35.7	58.5 ± 35.1	38.6 ± 33.9	0.007
**Connection issue delaying consultation via IMA**		Percentage	23.6 ± 19.8	28.2 ± 22.5	18.8 ± 15.4	0.02

**The current Consultation Request Tool is adequate**	Disagree	19 (17.9%)	10 (18.2%)	9 (17.6%)	0.26
	Neutral	39 (36.8%)	24 (43.6%)	15 (29.4%)	
	Agree	48 (45.3%)	21 (38.2%)	27 (52.9%)	
**The current Consultation Request Tool should be improved/more organized**	Disagree	2 (1.9%)	1 (1.8%)	1 (2.0%)	0.79
	Neutral	26 (24.5%)	15 (27.3%)	11 (21.6%)	
	Agree	78 (73.6%)	39 (70.9%)	39 (76.5%)	
**It is necessary to develop an electronic platform or an application exclusively for consultation request**	Disagree	18 (17.0%)	12 (21.8%)	6 (11.8%)	0.35
	Neutral	18 (17.0%)	8 (14.5%)	10 (19.6%)	
	Agree	70 (66.0%)	35 (63.6%)	35 (68.6)	

Table 5 presents participants opinion regarding the use of IMA for consultations. Text messaging was the most used consultation tool via IMA, with 95.6% of participants favoring it, and no significant difference between consultants and consultees (97.9% vs 93.2%, p = 0.28). Voice messages were also widely used (82.4%) by both consultants and consultees. Moreover, images were used by consultees more than consultants (63.6 % vs 42.6%, p = 0.04). In terms of trust, text messages remained the most trusted tool (70.3%). However, consultants were significantly more likely to trust image-based consultations than consultees (29.8% vs 4.5%, p=0.002). On the other hand, voice messages were trusted by 40.4% of consultants but only 8.6% of consultees, reflecting a significant difference (p = 0.001). Voice messages were reported as the most distrusted tool by 44.0% of participants, followed by images (24.2%), with no significant differences between groups. When rating the utility of IMA-based consultations on a range of 0% (Never) to 100% (Always), participants rated IMA as fast (84.4 ± 23.6), easy (87.9 ± 24.0), and accessible (88.5 ± 19.5), with no major differences between groups. However, consultants perceived IMA consultations as significantly more distracting than consultees (58.5 ± 35.1 vs 38.6 ± 33.9, p = 0.007).

Finally, 45.3% agree that the current consultation process is adequate, while 73.6% believe it should be improved or better organized, and two-thirds (66.0%) agree that an electronic platform or application should be developed for consultations.

Discussion

The study included 120 participants, with 65 consultants and 55 consultees. Consultants were older than consultees, and while all consultees were residents, 35.4% of consultants were fellows. The participants' residency programs varied significantly, with consultants more likely to belong to surgical specialties while none of the consultees were from surgical fields. Regarding consultation methods, all participants used SP while on duty, with IMAs being the most used. Consultants favored SP calls, whereas consultees relied more on workstation phone calls. Consultants also had significantly longer shift durations. Consultation patterns varied between day and night shifts, with consultees more likely to use pagers and workstation phone calls, while consultants preferred SP calls, particularly at night. WhatsApp was the most commonly used IMA, and text messaging was the most utilized consultation tool. Consultants trusted images and voice messages more than consultees. Overall, the study highlights significant differences in consultation preferences and patterns between consultants and consultees, particularly in communication methods.

Two key factors emerge as influential in the use of SPs for consultations: age and shift duration. The overall higher prevalence of SP uses among consultees compared to consultants can likely be attributed to their younger age, as younger healthcare professionals tend to be more comfortable utilizing digital communication tools in clinical practice(13). This aligns with previous studies showing that younger physicians are more open to adopting mobile applications for patient care and clinical decision-making.

In a study exploring smartphone and medical application usage in clinical settings across King Hamad University Hospital in Bahrain and Queen Mary Hospital in Hong Kong, 48% of junior doctors reported high smartphone reliance, compared to 32.3% of senior doctors(14). Furthermore, the preference for IMAs like WhatsApp suggests that younger professionals prefer asynchronous, text-based communication, which has been shown to enhance efficiency in clinical workflows. In fact, according to a study done by Menon R. et al. in 2025 the use of such asynchronous platforms improves clinical communication compared to synchronous methods, enhancing workflow efficiency and potentially leading to better patient care outcomes(15). Additionally, the higher use of SP among consultants compared to consultees is likely linked to their significantly longer shift durations.

Consultants, often more senior and tasked with overseeing patient care for an extended period, may rely more on SPs for rapid communication and coordination. The preference for phone calls via SP among consultants compared to consultees may reflect the need for immediate responses, particularly during critical decision-making. Previous research has highlighted that longer shifts and heavier workloads contribute to increased mobile device usage in healthcare settings, as these tools provide quick access to information and facilitate rapid consultations. A study in Kuwait found that smartphone use was widespread among surgeons during the pandemic, valuing it for

improving communication, literature review, and clinical decision-making(16). The preference for SPs among consultants highlights the importance of healthcare institutions considering policies and infrastructure that support secure and efficient mobile communication, ensuring both the effectiveness of clinical operations and the confidentiality of patient information.

Clear differences emerge in consultation and communication patterns between consultants and consultees. This observed difference in consultation patterns highlights variations in communication preferences and accessibility. Consultees demonstrated a greater reliance on pagers and workstation phone calls, particularly during night shifts. This could be attributed to their structured workflow and hierarchical nature of consultations, where they often initiate contact through formal channels. In contrast, consultants favored personal SP calls at night, likely due to their increased autonomy and need for immediate decision-making in critical situations. Moreover, consultants may not notice a consultation via IMA when on call during night shift, hence they might rely more on calls. Despite the widespread use of IMAs across both groups, consultees conducted a higher proportion of consultations through IMAs during both day and night calls, though this difference was not statistically significant. This trend aligns with previous findings suggesting that younger healthcare professionals are more inclined to use asynchronous communication for efficiency (15). WhatsApp emerged as the dominant IMA for consultations, with nearly universal adoption among participants, according to a study done by Mars M. et al. WhatsApp is recognized as an easy-to-use, cost-effective (free of charge), and efficient communication tool, which explains its increasing use in healthcare sector (13). Consultants also reported continued engagement in IMA-based consultations beyond working hours. While IMAs such as WhatsApp enhance the speed, accessibility, and efficiency of clinical consultations, they also present significant cybersecurity challenges. The transmission and storage of sensitive patient information on personal mobile devices, outside of secure hospital networks, increases the risk of data breaches and unauthorized access. Such vulnerabilities not only compromise patient confidentiality but also expose healthcare providers and institutions to serious medico-legal consequences, underscoring the need for robust cybersecurity measures and stricter regulatory oversight in clinical communication practices.

It is evident that text messaging remains the predominant consultation tool via Instant Messaging Applications (IMA), favored by 95.6% of participants, reinforcing its role as a rapid and convenient mode of communication in clinical settings. Voice messages were also widely utilized, while consultees relied more on image-based consultations compared to consultants, possibly due to their need for visual confirmation in decision-making. However, trust levels varied across different modalities, with consultants exhibiting significantly greater trust in image-based and voice message consultations than consultees. Notably, voice messages were the most distrusted format, likely due to concerns about misinterpretation or lack of clarity (17). The perceived advantages of IMA-based consultations were highlighted, with participants rating them highly in terms of speed, ease of use, and accessibility. In a study done in 2021, healthcare workers at the Emergency department found the use of SP helpful in coordination among healthcare providers, contributing positively to patient care (18). However, consultants were significantly more likely to find these consultations distracting, possibly due to their greater responsibility and

workflow interruptions. Additionally, challenges such as connection issues and diagnostic errors were more frequently reported by consultants (who circulates more in the hospital when compared to ED staff who remain in the ED), indicating a need for improved infrastructure and training on the limitations of remote communication.

Finally, the demand for a structured and secure electronic consultation platform was also notable, with two-thirds of respondents supporting its development. These findings highlight the need for standardized guidelines and dedicated digital platforms to enhance the efficiency, security, and reliability of remote clinical communication. Morris C. et al, showed that despite WhatsApp's widespread use, clinicians often neglect or fail to report proper record-keeping of consultations, with no established best practices for securing patient data, hence, raising awareness and providing clear guidelines are urgently needed(18).

In summary, this study reveals significant variations in communication methods and consultation patterns between consultants and consultees, with personal smartphones and Instant Messaging Applications, particularly WhatsApp, being heavily utilized. While these tools enhance communication efficiency, they also raise concerns regarding data security and patient confidentiality. This study highlights the need for more structured and secure digital solutions to support the consultation process. Previous studies highlight the need for legal regulations on the use of IMAs for consultations and the development of applications solely for medical purposes, as patient confidentiality is at risk (30). To address these gaps, we propose the development of an AI-generated application tailored to hospital consultations. Such an app could standardize and streamline communication, enhance data security, and ensure compliance with privacy regulations, ultimately improving the efficiency and safety of the consultation process in clinical settings. This approach could bridge the existing knowledge gap by integrating modern technology with healthcare communication, providing a more secure and reliable platform for consultations.

Limitations

This study has several limitations. First, the cross-sectional design limits the ability to establish causal relationships or assess changes over time. The study is also restricted to a single institution, AUBMC, with a small sample size of only 120 participants, which may limit the generalizability of the findings to other settings or larger, more diverse populations. Furthermore, attending physicians were not included in the participant population, which may affect the comprehensiveness of the findings regarding communication dynamics. In fact, some ED attendings physicians who may opt to contact the attending consultant directly without going through the channel of residents

Moreover, some consultations are conducted in person when a consultant is already present and evaluating another patient. Rather than utilizing formal communication methods such as IMA or phone calls, ED physicians often choose face-to-face interactions with consultants to inform them of additional consults. While this direct interaction facilitates immediate discussion, faster decision-making, and more efficient patient management, it may reduce the potential for delays associated with electronic or phone-based communication.

Finally, the study did not explore whether participants had used other mobile applications prior to using the WhatsApp for consultations, which may have influenced their preferences and attitudes toward WhatsApp.

DECLARATION

Funding: not applicable

Data sharing: Data are available on reasonable request.

Competing interest's declaration: none to be declared.

Clinical trial number: not applicable

Ethics approval and consent to participate: This study was conducted in accordance with the ethical standards of the institutional research committee and with the 1964 Helsinki Declaration and its later amendments or comparable ethical standards. This study was approved by the Institutional Review Board of the American University of Beirut (SBS-2023-0009). Electronic informed consent was obtained via Lime Survey. Participation was voluntary, anonymous, and no identifying data were collected.

Consent to participate: All participants provided informed electronic consent via LimeSurvey before completing the survey.

Consent for publication: Not available

Acknowledgement: Not applicable

References

1. Maassen O, Fritsch S, Gantner J, Deffge S, Kunze J, Marx G, et al. Future mobile device usage, requirements, and expectations of physicians in German university hospitals: web-based survey. Journal of medical Internet research. 2020;22(12):e23955.
2. Boruff JT, Storie D. Mobile devices in medicine: a survey of how medical students, residents, and faculty use smartphones and other mobile devices to find information. Journal of the Medical Library Association: JMLA. 2014;102(1):22.
3. Mosa ASM, Yoo I, Sheets L. A systematic review of healthcare applications for smartphones. BMC medical informatics and decision making. 2012;12:1-31.
4. Divall P, Camosso-Stefinovic J, Baker R. The use of personal digital assistants in clinical decision making by health care professionals: a systematic review. Health informatics journal. 2013;19(1):16-28.
5. Gaj F, Peracchini M, Passannanti D, Quaresima S, Giovanardi F, Lai Q. Use of telemedicine in the postoperative assessment of proctological patients: a case–control study. Techniques in coloproctology. 2023;27(2):153-8.
6. Wu RC, Morra D, Quan S, Lai S, Zanjani S, Abrams H, et al. The use of smartphones for clinical communication on internal medicine wards. Journal of Hospital Medicine. 2010;5(9):553-9.
7. Soto RG, Chu LF, Goldman JM, Rampil IJ, Ruskin KJ. Communication in critical care environments: mobile telephones improve patient care. Anesthesia & Analgesia. 2006;102(2):535-41.
8. Ortega GR, Taksali S, Smart R, Baumgaertner MR. Direct cellular vs. indirect pager communication during orthopaedic surgical procedures: a prospective study. Technology and Health Care. 2009;17(2):149-57.
9. O'Connor C, Friedrich JO, Scales DC, Adhikari NK. The use of wireless e-mail to improve healthcare team communication. Journal of the American Medical Informatics Association. 2009;16(5):705-13.
10. Gulacti U, Lok U. Comparison of secure messaging application (WhatsApp) and standard telephone usage for consultations on Length of Stay in the ED. Applied clinical informatics. 2017;8(03):742-53.
11. Hitti E, Hadid D, Melki J, Kaddoura R, Alameddine M. Mobile device use among emergency department healthcare professionals: prevalence, utilization and attitudes. Scientific Reports. 2021;11(1):1917.
12. Alameddine M, Soueidan H, Makki M, Tamim H, Hitti E. The use of smart devices by care providers in emergency departments: cross-sectional survey design. JMIR mHealth and uHealth. 2019;7(6):e13614.
13. Mars M, Scott RE. WhatsApp in clinical practice: a literature review. The Promise of New Technologies in an Age of New Health Challenges. 2016:82-90.
14. Nair AA, Afroz S, Ahmed BU, Ahmed UU, Foo CC, Zaidan H, et al. Smartphone usage among doctors in the clinical setting in two culturally distinct countries: cross-sectional comparative study. JMIR mHealth and uHealth. 2021;9(5):e22599.
15. Jhala M, Menon R. Examining the impact of an asynchronous communication platform versus existing communication methods: an observational study. BMJ innovations. 2021;7(1).

16. Buabbas AJ, Aldousari S, Ayed AK, Safar M, Alkandari O. Usefulness of smartphone use among surgeons in clinical practice during the pandemic of COVID-19: a cross-sectional study. BMC Medical Informatics and Decision Making. 2021;21(1):198.
17. Blom L, Laflamme L, Mölsted Alvesson H. Expectations of medical specialists about image-based teleconsultation–A qualitative study on acute burns in South Africa. PLoS One. 2018;13(3):e0194278.
18. Morris C, Scott RE, Mars M. WhatsApp in clinical practice—the challenges of record keeping and storage. A scoping review. International journal of environmental research and public health. 2021;18(24):13426.
