# Supplementary material for: Using instant messaging applications for consultations in the emergency department: A cross-sectional survey
Source: PLoS One. 2026 Apr 15;21(4):e0347028. doi: 10.1371/journal.pone.0347028 (PMC13082625; doi:10.1371/journal.pone.0347028)
Supplement: Attachment — Submitted filename: Response_to_Reviewers_auresp_2.docx [file pone.0347028.s006.docx]

PLOS ONE PONE-D-25-46511Point by point response

| Comment | Response |
| --- | --- |
| Study Design |  |
| 1. The second sentence is redundant and can be removed. “This is a cross-sectional study conducted at..” | Thank you for the comment. The sentence has been removed and the paragraph was revised to improve clarity and conciseness. |
| 2. In the methods section that describes the consulting process, the authors mention that and order is placed followed by the ED team contacting the consultant team. What is the goal of this second contact? Is this where patient information is shared and the question to the consultant is outlined? Or is that done through the order? Please clarify.. | Thank you for the comment. We revised the Methods section to clarify the consultation workflow. Specifically, we clarified that a consultation order is placed in the EMR for documentation and tracking purposes but does not generate an automatic notification to the consulting team. The ED team subsequently contacts the on-call consultant directly using any method of the ones mentioned in the manuscript to communicate patient information and the clinical question. |
| Table 2 |  |
| What does use of one’s smartphone by someone else mean? | Thank you for the comment. We clarified the wording of this variable in Table 2. It refers to situations in which another team member uses the participant’s smartphone to communicate with consulting services during clinical duties. |
